# Dublin Dissector—Fifth Edition

**Published:** 1848-06

**Authors:** 


DUBLIN DISSECTOR—FIFTH EDITION.

We have been somewhat remiss in not calling attention to the new edition of this admirable book. The author, Dr. Harrison, has given it a thorough revision, introduced numerous engravings, and added much new matter, particularly on General and Structural Anatomy, also on the Nervous System and on the Organs of Sense. These additions increased the size so much as to render it expedient to divide the work into two volumes, each of which has a copious index. In its present form it is one of the most beautiful books that we have ever seen.

